# Supplementary figures and images for: Deep Isolated Aquifer Brines Harbor Atypical Halophilic Microbial Communities in Quebec, Canada
Source: Genes (Basel). 2023 Jul 26;14(8):1529. doi: 10.3390/genes14081529 (PMC10454208; doi:10.3390/genes14081529)

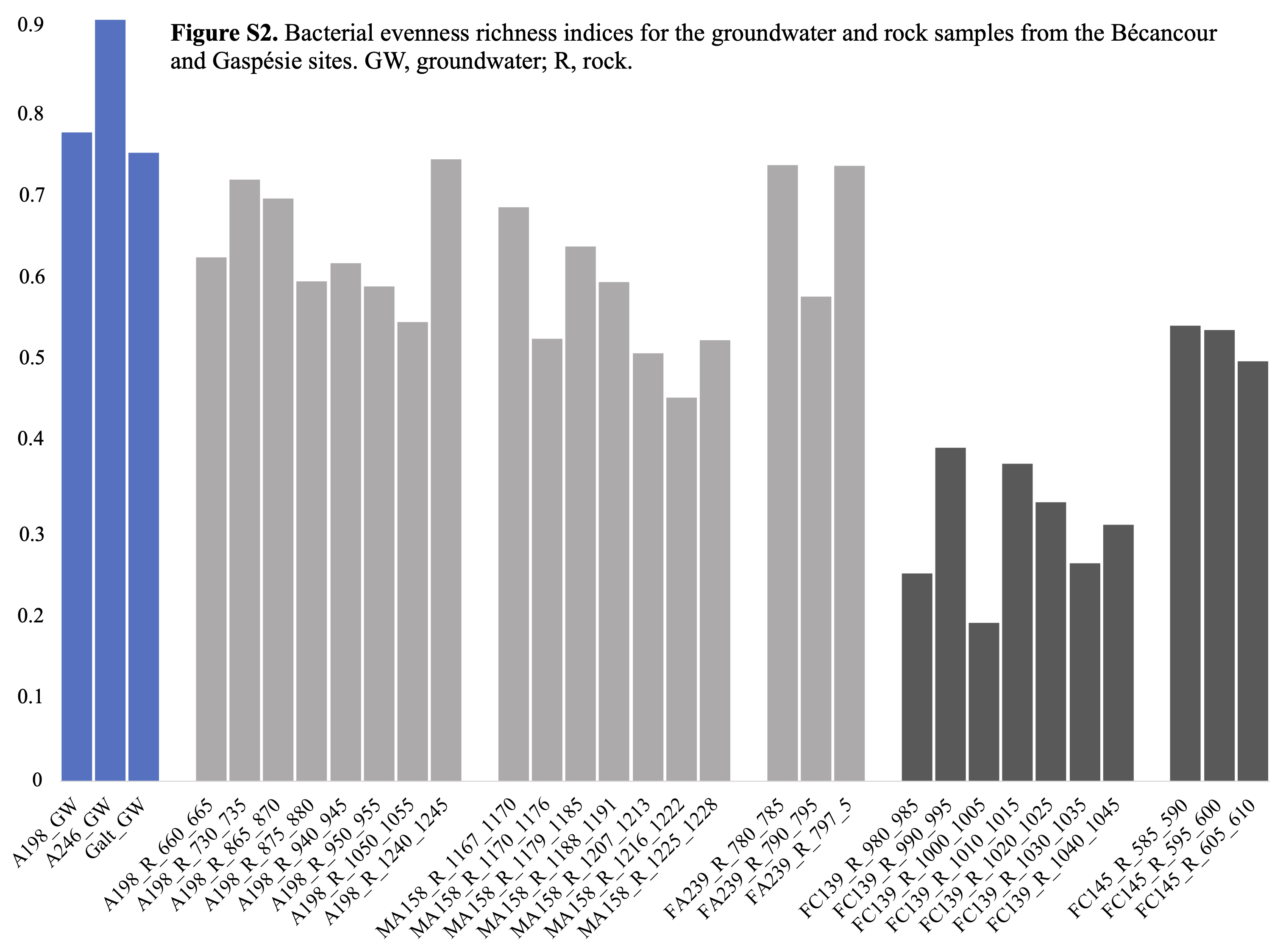

Supplement: Supplementary file 1 [file genes-14-01529-s001.zip › Supplementary Material/Figure S2.tiff]

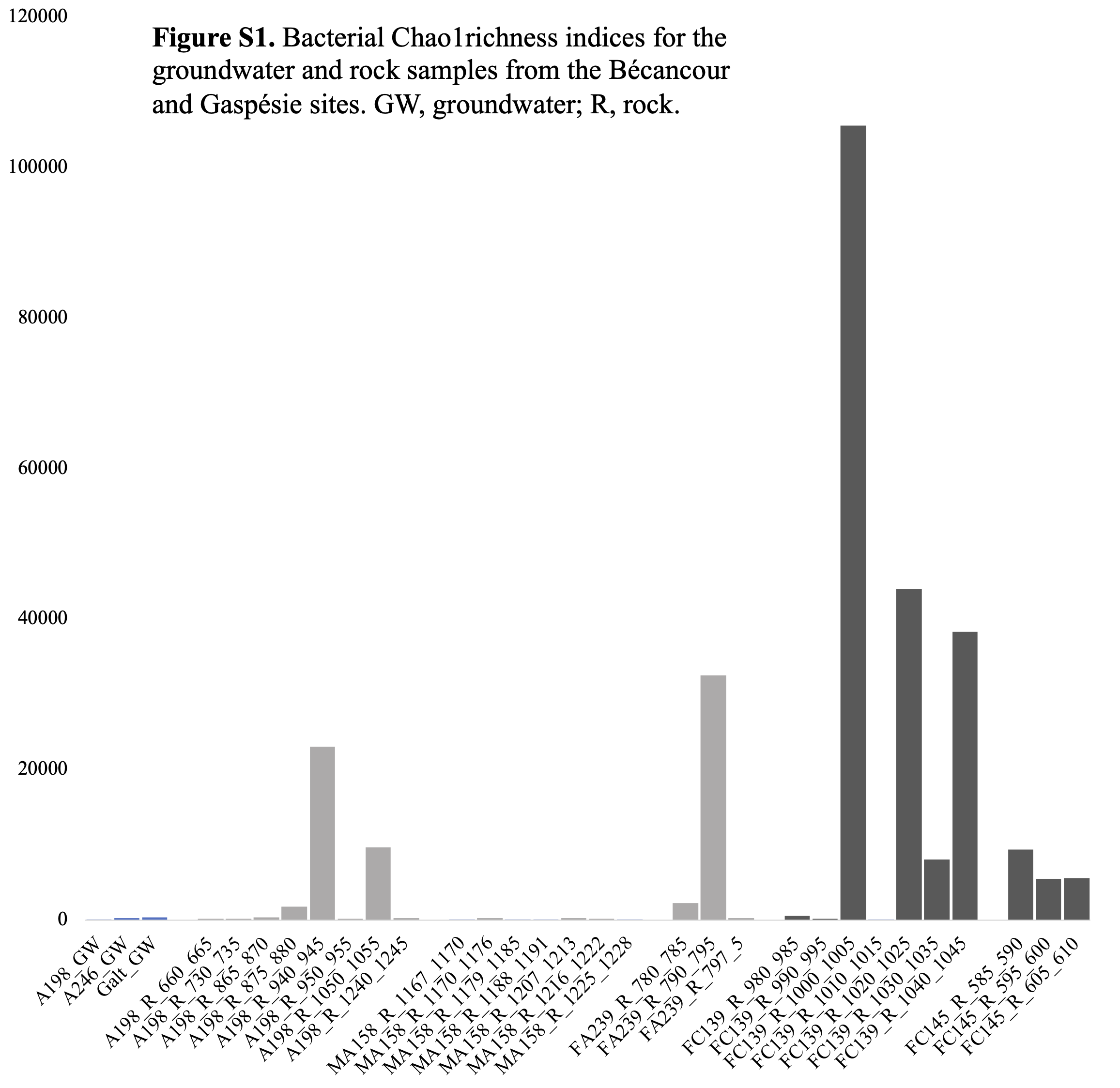

Supplement: Supplementary file 1 [file genes-14-01529-s001.zip › Supplementary Material/Figure S1.tiff]
